# Long-Term miRNA Changes Predicting Resiliency Factors of Post-Traumatic Stress Disorder in a Large Military Cohort—Millennium Cohort Study

**DOI:** 10.3390/ijms26115195

**Published:** 2025-05-28

**Authors:** Ruoting Yang, Swapna Kannan, Aarti Gautam, Teresa M. Powell, Cynthia A. LeardMann, Allison V. Hoke, George I. Dimitrov, Marti Jett, Carrie J. Donoho, Rudolph P. Rull, Rasha Hammamieh

**Affiliations:** 1 Medical Readiness Systems Biology, Walter Reed Army Institute of Research, Silver Spring, MD 20910, USA; swapna.kannan2.ctr@health.mil (S.K.); aarti.gautam.civ@health.mil (A.G.); allison.v.hoke.civ@health.mil (A.V.H.); george.i.dimitrov.ctr@health.mil (G.I.D.); marti.jett-tilton.civ@health.mil (M.J.); rasha.hammamieh1.civ@health.mil (R.H.); 2 Army Resilience Directorate, HQDA DCS G-1, Arlington, VA 22201, USA; teresa.m.powell14.ctr@army.mil; 3 Deployment Health Research Department, Naval Health Research Center, San Diego, CA 92152, USA; cynthia.a.leardmann.ctr@health.mil (C.A.L.); rudolph.p.rull2.civ@health.mil (R.P.R.); 4 Leidos, Inc., San Diego, CA 92106, USA; 5 Army Analytics Group, Research Facilitation Laboratory, Monterey, CA 93940, USA; carrie.j.donoho.mil@army.mil

**Keywords:** miRNA sequencing, PTSD, military, resilience

## Abstract

Post-traumatic stress disorder (PTSD) is a complex, debilitating condition prevalent among military personnel exposed to traumatic events, necessitating biomarkers for early detection and intervention. Using data from the Millennium Cohort Study, the largest and longest-running military health study initiated in 2001, our objective was to identify specific microRNA (miRNA) expression patterns associated with distinct PTSD symptom trajectories among service members and veterans and assess their potential for predicting resilience and symptom severity. We analyzed 1052 serum samples obtained from the Department of Defense Serum Repository and linked with survey data collected at baseline and across three follow-up waves (2001–2011), using miRNA sequencing and statistical modeling. Our analysis identified five PTSD trajectories—resilient, pre-existing, new-onset moderate, new-onset severe, and adaptive—and revealed significant dysregulation of three key miRNAs (miR-182-5p, miR-9-5p, miR-204-5p) in participants with PTSD compared to resilient individuals. These miRNAs, which inhibit brain-derived neurotrophic factor (BDNF) and target pathways like NFκB, Notch, and TGF-alpha, were associated with neuronal plasticity, inflammation, and tissue repair, reflecting PTSD pathophysiology. These findings suggest that miRNA profiles could serve as biomarkers for early identification of PTSD risk and resilience, guiding targeted interventions to improve long-term health outcomes for military personnel.

## 1. Introduction

Post-traumatic stress disorder (PTSD) represents a significant public health concern that is characterized by debilitating symptoms and considerable morbidity [1,2]. Military personnel have a heightened risk of developing PTSD due to the nature of their work that may expose them to traumatic events and life-threatening situations such as combat [3]. Understanding the molecular underpinnings of PTSD trajectories and associated comorbidities, such as depression, substance misuse, and sleep disorder, is essential to early detection of PTSD symptoms that will enable improved long-term health outcomes for service members [4].

MicroRNAs (miRNAs) offer a promising avenue for understanding the biological underpinnings of PTSD. miRNAs are short, noncoding sequences that regulate gene expression by suppressing protein-coding mRNA, a process known as epigenetic regulation, without altering the DNA structure. Many animal studies indicate that miRNAs play a significant role in the molecular mechanisms underlying fear memory formation and stress responses associated with PTSD, particularly those related to the hypothalamic–pituitary–adrenal (HPA) axis [5,6]. Evidence also suggests that exposure to traumatic stress induces altered miRNA expression in key brain regions implicated in PTSD, such as the prefrontal cortex (PFC) and amygdala [7,8]. It also influences inflammation by modulating proinflammatory T cells and cytokine profiles, contributing to the chronic inflammation often observed in PTSD [9]. Human clinical studies on miRNA in PTSD are limited. Lee et al. performed miRNA profiling in whole plasma, extracellular vesicles (EV), and EV-depleted plasma (EVD) samples from 24 male Iraq and Afghanistan combat veterans with and without PTSD using next-generation sequencing. They found that the overall miRNA profiles were different in whole plasma, EV, and EVD fractions and that miRNAs affected by PTSD were also distinct in each sample type. Specifically, miR-203a-3p in EV and miR-339-5p in EVD were significantly altered in veterans with PTSD [10]. Another study found that miRNAs linked to immune function and inflammation were consistently associated with PTSD [11]. The miRNA studies focused on cross-sectional comparisons between PTSD and resilient controls, offering insights into the molecular differences associated with PTSD. However, understanding the trajectories of PTSD symptomatology over time can help elucidate the dynamic changes in tandem with symptom development and identify actionable resilience factors [12].

The Millennium Cohort Study, initiated in 2001, is the largest and longest-running military health study with prospective follow-up of enrolled service members [13,14] and offers a unique opportunity to examine potential vulnerabilities, exposures, and symptoms as they emerge. Serum samples previously collected from Millennium Cohort participants were obtained from the Department of Defense Serum Repository (DoDSR) through the Armed Forces Health Surveillance Division (AFHSD). Our objective was to identify specific miRNA patterns associated with different PTSD trajectories and assess their potential for predicting alterations in self-reported PTSD symptoms.

## 2. Results

This study protocol was approved by the Naval Health Research Center Institutional Review Board in compliance with all applicable federal regulations governing the protection of human subjects. Serum specimens were obtained from the Department of Defense Serum Repository: The Armed Forces Health Surveillance Division, Defense Health Agency, U.S. Department of Defense, Silver Spring, Maryland (years of serum specimens: 2003–2012; serum specimens released: 2015–2017). Research data were derived from approved Naval Health Research Center Institutional Review Board protocol numbers NHRC.2014.0013 and NHRC.2000.0007 and approved by Walter Reed Army Institute of Research (WRAIR) protocol number WRAIR2775. All participants provided written or electronic informed consent in accordance with the study protocol approved by the Naval Health Research Center Institutional Review Board (initial consent was obtained between 2001 and 2003 and consent to use biospecimens between 2014 and 2015).

**Figure 1:**
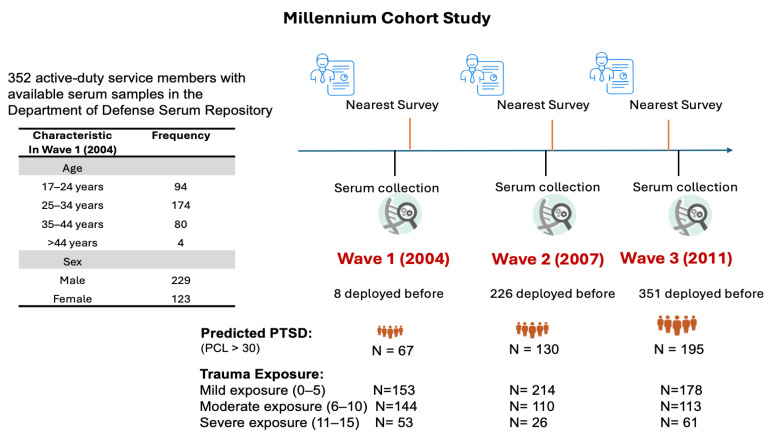
Study design in the Millennium Cohort Study. This schematic illustrates the alignment of serum sample collection (*n* = 352 participants) from the Department of Defense Serum Repository (DoDSR) with Millennium Cohort Study survey waves (Wave 1: 2004; Wave 2: 2007; Wave 3: 2011) for active-duty U.S. military personnel. The timeline depicts the first deployment distribution (x-axis) and survey wave timing (vertical dashed lines), with serum sampling points indicated. Accompanying tables summarize baseline demographics (age categories, sex distribution), deployment status, predicted PTSD status (PCL-C score > 30, indicating probable PTSD), and trauma exposure (scored 0–3 based on self-reported trauma frequency). These data contextualize the analysis of PTSD symptom trajectories and associated miRNA expression profiles over time.

Figure 1 illustrates the study design and participant deployment timeline, showing the distribution of participants based on their first deployment time and aligning serum collection with survey waves to identify molecular differences between active-duty personnel who developed PTSD and those who remained resilient. Age and sex were assessed at the time of baseline survey completion, while trauma exposure reflects cumulative exposure to combat and traumatic stressors assessed via self-reported responses to five questions on personal trauma experiences, including witnessing death due to war or disaster, physical abuse, decomposing bodies, maimed individuals, or prisoners/refugees, scored on a scale of 0 (none) to 3 (more than once).

**Figure 2:**
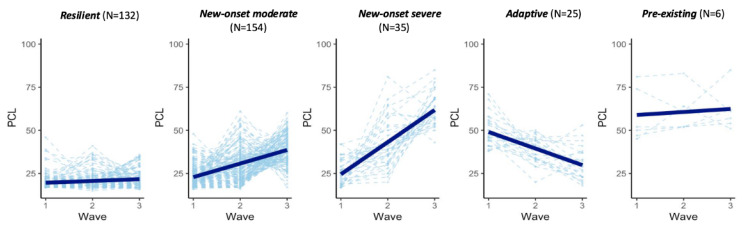
The trajectories of PTSD symptom severity (PCL-C scores) over three waves were clustered in five response groups: resilient (N = 132), new-onset moderate (N = 154), new-onset severe (N = 35), adaptive (N = 25), and pre-existing (N = 6). This plot illustrates five distinct PTSD symptom trajectories identified via latent growth mixture modeling of PCL-C scores (range: 17–85) across three waves (2004–2011) in the Millennium Cohort Study. Trajectories include resilient (N = 132, consistently low scores), new-onset moderate (N = 154, gradual increase post-deployment), new-onset severe (N = 35, sharp rise post-deployment), adaptive (N = 25, moderate baseline scores decreasing over time), and pre-existing (N = 6, persistently high scores). Bold blue lines depict group mean PCL-C scores at each wave, while faint lines trace individual participant trajectories, highlighting heterogeneity in symptom progression.

Figure 2 presents age and sex distribution of participants at Wave 1 (2004–06). Additional characteristics, including deployment status, predicted PTSD status, and trauma exposure levels (mild, moderate, severe) across the waves are displayed. These characteristics provide essential context for analyzing PTSD symptom trajectories and miRNA profiles.

Table 1 presents a comparison of demographic and clinical variables between control and PTSD groups across three waves. Statistically significant differences were observed in PHQ scores, PCL scores, and sleep duration, with consistently higher symptom burden and shorter sleep in the PTSD group.

We first revealed distinct trajectories of PTSD symptoms among participants and then examined the association between miRNA profiles and distinct PTSD symptom trajectories.

### 2.1. Five Trajectories of PTSD Symptoms

Using latent growth mixture modeling on PCL-C scores, we identified five distinct trajectories of PTSD symptoms among participants: resilient, new-onset moderate, new-onset severe, adaptive, and pre-existing (Figure 2). Each trajectory displayed unique patterns in the progression of PTSD symptoms over the study period:

Resilient (N = 132): this group maintained consistently low levels of PTSD symptoms throughout the study, demonstrating strong resilience in coping with stressors.

New-onset moderate (N = 154): participants in this group initially exhibited low levels of PTSD symptoms but experienced a moderate increase in symptoms following deployment and exposure to stressors.

New-onset severe (N = 35): similar to the new-onset moderate group, participants initially showed low levels of PTSD symptoms. However, post-deployment, they experienced a significant and severe rise in symptom severity.

Adaptive (N = 25): this group displayed moderate levels of PTSD symptoms at the start, which gradually decreased over time. These participants may have experienced prior trauma or stressors but showed a reduction in symptoms over the course of the study.

Pre-existing (N = 6): participants in this group had high levels of PTSD symptoms prior to deployment, and these symptoms persisted without significant change over time. This group reflects individuals who had already developed PTSD symptoms before the study period, which were sustained over the course of the study.

Each trajectory underscores different experiences and responses to stressors, highlighting the heterogeneity in PTSD symptom development and recovery.

### 2.2. miRNA Associated with Changes in PTSD Symptoms

To analyze the relationship between miRNAs and PTSD symptom severity (assessed using PTSD Checklist-Civilian Version [PCL-C] scores), we used a linear mixed-effects model. After adjusting for age, sex, deployment history, and trauma exposure and accounting for within-subject variability over time, we identified several miRNAs associated with different PTSD trajectories.

Specifically, five miRNAs (miR-10a-5p, miR-320b, miR-1246, miR-181b-5p, let-7c-5p) and 31 miRNAs were associated with new-onset severe PTSD and moderate PTSD (*p* < 0.05). Additionally, miR183-5p showed an association with an adaptive trajectory.

### 2.3. Comparative Analysis of miRNA Expression Patterns Across PTSD Trajectories

To further elucidate the role of miRNAs with PTSD changes over time, we conducted a comparative analysis of their expression patterns across distinct response trajectories. This involved pairwise comparisons between specific trajectories to identify miRNAs that exhibit significant variations in expression levels.

#### 2.3.1. Adaptive vs. Resilient Trajectories

**Figure 3:**
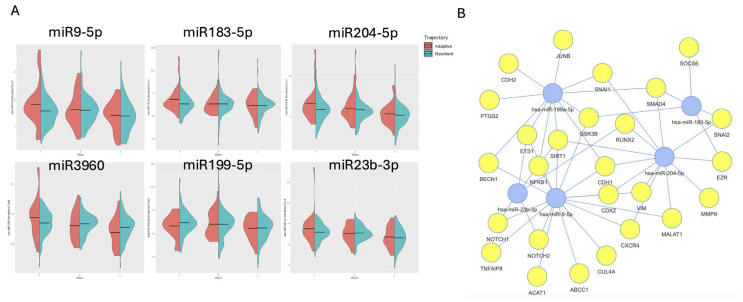
Differential miRNA expression and pathway enrichment in adaptive vs. resilient PTSD trajectories. (**A**) Violin plots showing expression levels (logCPM) of significant miRNAs (e.g., miR-9-5p, *p* = 0.001; miR-204-5p, *p* = 0.003; miR-23b-3p, *p* = 0.008) in adaptive (red, N = 25) and resilient (blue, N = 132) groups across waves. Adaptive participants exhibit higher baseline expression (Wave 1), converging toward resilient levels by Wave 3, reflecting temporal adaptation. Width indicates expression density; medians are marked. (**B**) Network analysis of miRNA targets highlighting enrichment in Notch signaling and TGF-alpha pathways. Blue nodes represent miRNAs, and yellow nodes represent target genes.

We first compared miRNA expression profiles between individuals demonstrating an adaptive response (N = 25) and those exhibiting resilience (N = 132). The accompanying Figure 3 illustrates the expression patterns of the most significant miRNAs identified in this comparison. Each panel represents a specific miRNA, with violin plots depicting the distribution and density of its expression within each group (Figure 3A). These visualizations reveal distinct temporal patterns in miRNA expression between adaptive and resilient groups. In Wave 1, most of the identified miRNAs exhibit significantly higher expression in the adaptive group compared to the resilient group. However, this difference diminishes over time, with expression levels gradually becoming more similar between the two groups. Importantly, these significant miRNAs, such as miR-9-5p, miR-204-5p, and miR-23b-3p, target several essential genes, including NFkB and NOTCH, which are key components of pathways involved in cellular stress response, inflammation, and cell fate decisions. Pathway enrichment analysis further indicates that these miRNAs are significantly enriched in the TGF-alpha pathway, which is known to play a role in tissue repair and regeneration, as well as the NFkB and Notch signaling pathways (Figure 3B).

#### 2.3.2. New-Onset vs. Resilient Trajectories

Subsequently, we compared miRNA expression patterns between individuals with a new-onset moderate PTSD (N = 134), those in the new-onset severe PTSD group (N = 35), and those in the resilient group (N = 132). The number of significant miRNA was generally less than the previous comparison. Figure S1A highlights two representative miRNAs (miR141-3p and miR182-5p) that are significantly downregulated in the new-onset group (red) compared to the resilient group (blue). Network analysis of all differentially expressed miRNAs identified a targeted gene network focused on circadian genes CLOCK and BDNF, a key regulator of neuroplasticity and neuronal survival implicated in the pathophysiology of PTSD (Figure S1B).

### 2.4. Differential Analysis Between PTSD Positive and Negative

In our differential analysis, we compared the miRNA profiles between 392 PTSD samples and 660 control samples without PTSD, determined using the PCL-C with an initial cutoff of 30, reflecting *Diagnostic and Statistical Manual of Mental Disorders, Fourth Edition (DSM-IV)* criteria for probable PTSD. Using robust statistical methods and stringent multiple testing correction, we found three significant differentially expressed (DE) miRNAs (false discovery rate (FDR) < 0.05 and |log-fold change| > 0.4) (Table 2). When the PCL-C cutoff was increased to 38, these three DE-miRNAs remained significant.

All three differentially expressed miRNAs (DEmiRNAs) identified between PTSD and control groups—hsa-miR-204-5p, hsa-miR-9-5p, and hsa-miR-182-5p—also exhibited significant sex-specific expression patterns. Notably, the expression levels of these miRNAs were consistently lower in females compared to males, suggesting a potential sex-based regulatory mechanism underlying their association with PTSD. 

**Figure 4:**
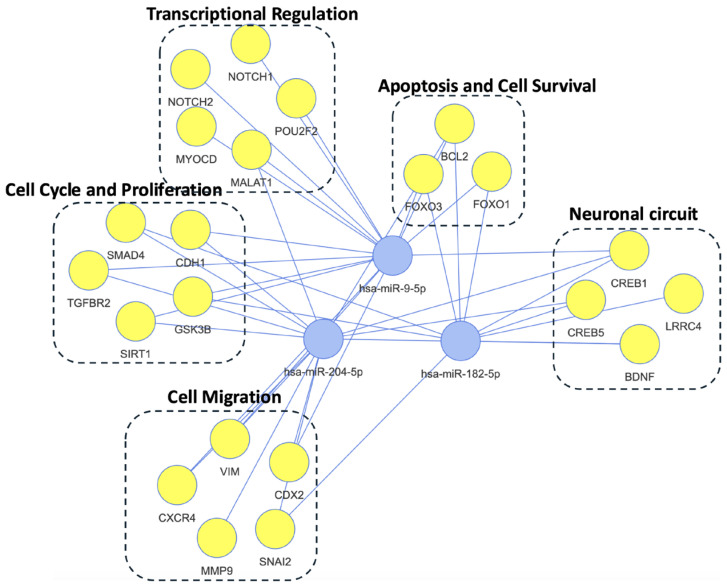
Regulatory network of three PTSD-associated miRNAs and their target genes. This network visualizes the interactions of three differentially expressed miRNAs (hsa-miR-9-5p, hsa-miR-182-5p, hsa-miR-204-5p) with their experimentally validated target genes, identified via miRTarBase, in 392 PTSD-positive vs. 660 PTSD-negative samples. Genes are grouped into five functional categories: Transcriptional Regulation (e.g., NOTCH1, MYOCD), Apoptosis and Cell Survival (e.g., BCL2, FOXO3), Cell Migration (e.g., VIM, MMP9), Neuronal Circuit (e.g., BDNF, CREB1), and Cell Cycle and Proliferation (e.g., SMAD4, GSK3B). miRNAs (blue nodes) connect to targets (yellow nodes), illustrating their pleiotropic roles in PTSD pathophysiology, including neuroplasticity and inflammation.

This network analysis in Figure 4 illustrates the complex interplay between three DE miRNAs (hsa-miR-9-5p, hsa-miR-182-5p, and hsa-miR-204-5p) and their target genes, which are grouped into five functional categories. The “Transcriptional Regulation” category features transcription factors like NOTCH1, NOTCH2, POU2F2, and MYOCD, alongside the long non-coding RNA MALAT1, all crucial for modulating gene expression and cellular differentiation. Genes involved in apoptosis regulation and cell survival, such as FOXO1, FOXO3, and BCL2, comprise the “Apoptosis and Cell Survival” category. The “Cell Migration” category encompasses genes like VIM, SNAI2, CXCR4, CDX2, CDH1, and MMP9, which contribute to cell motility and tissue organization. Essential for neuronal function, synaptic plasticity, and signal transduction, genes like LRRC4, BDNF, CREB1, CREB5, and CACNA1C constitute the “Neuronal Circuit” category. Lastly, the “Cell Cycle and Proliferation” category comprises SMAD4, GSK3B, SIRT1, CDC42, and LDLRAP1, genes involved in cell growth, division, and diverse signaling pathways. This network underscores the pleiotropic roles of these miRNAs and their potential to influence cellular processes by regulating the expression of key genes involved in a wide array of biological functions.

Second, we compared pre-deployment miRNA expression profiles of participants who developed PTSD after deployment with those who remained resilient (PTSD-negative) throughout the study. Among participants who were negative for PTSD at pre-deployment, 208 individuals eventually developed probable PTSD following deployment, while 100 individuals maintained low PTSD symptom levels, demonstrating resilience. To identify DE miRNAs, we performed a negative binomial generalized linear model to compare miRNA expression between the groups, adjusting for potential confounders such as age, sex, and trauma exposure. After applying multiple testing correction with an FDR-adjusted *p*-value threshold of <0.05 and a |log-fold change| > 0.4, we identified four significant DE miRNAs, as presented in Table 3. This analysis highlights potential biomarkers of PTSD vulnerability and resilience, offering insights into pre-deployment risk profiles.

Among the four differentially expressed miRNAs, only hsa-miR-194-5p showed a significant sex difference, with higher expression in females compared to males.

**Figure 5:**
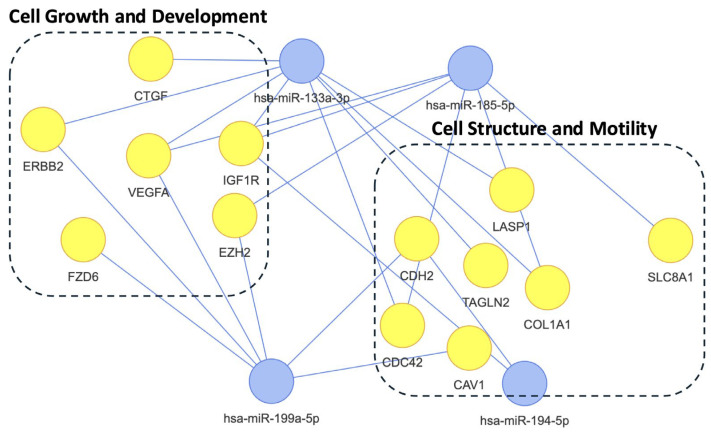
Pre-deployment miRNA network predicting PTSD vulnerability. This network depicts the regulatory interactions of four differentially expressed miRNAs (hsa-miR-185-5p, hsa-miR-194-5p, hsa-miR-199a-5p, hsa-miR-133a-3p) identified in pre-deployment serum samples from participants who later developed PTSD (N = 208) versus those who remained resilient (N = 100). Target genes, validated via miRTarBase, are categorized into two functional groups: Cell Growth and Development (e.g., CTGF, VEGFA, logFC range: −0.45 to −0.54) and Cell Structure and Motility (e.g., CDH2, CDC42). miRNAs (blue nodes) link to targets (yellow nodes), with SLC8A1 (ion transport) also targeted, suggesting roles in systemic vulnerability to PTSD-related cellular processes.

This network analysis highlights the regulatory roles of three microRNAs (miRNAs) on target genes grouped into two main functional categories (Figure 5). The first, “Cell Growth and Development”, includes genes like CTGF, ERBB2, VEGFA, IGF1R, FZD6, and EZH2, which are key players in proliferation, differentiation, and signaling pathways. The second, “Cell Structure and Motility”, features genes such as CDH2, COL1A1, CAV1, LASP1, TAGLN2, and CDC42, involved in adhesion, migration, and cytoskeletal dynamics. Additionally, SLC8A1, critical for ion transport and neuronal function, is part of the network. These miRNAs likely coordinate complex regulatory networks influencing fundamental processes in growth, development, structure, and motility.

## 3. Discussion

This study identified distinct PTSD symptom trajectories among service members and veterans and profiled miRNA expression across these trajectories, expanding on prior research by uncovering dynamic changes in miRNAs associated with symptom severity and recovery. Many participants developed PTSD symptoms following exposure to stressors. While some demonstrated resilience (“resilient”), a substantial portion experienced moderate (“new-onset moderate”) or severe (“new-onset severe”) increases in symptoms. Our analysis revealed notable differences in miRNA expression patterns between individuals with adaptive and resilient PTSD trajectories. At wave 1, the adaptive group exhibited significantly higher expression of several miRNAs, which gradually converged with the expression levels observed in the resilient group over time. These differentially expressed miRNAs target key genes such as NFκB and NOTCH, which are well-established regulators of the neurobiological stress response.

The Notch signaling pathway has been shown to regulate fear memory, amygdala reactivity, and synaptic plasticity—hallmarks of PTSD neurocircuitry—while also modulating inflammatory and stress hormone signaling. Dysregulation of this pathway may sensitize individuals to trauma-induced neuronal dysfunction [15]. Similarly, NFκB is a master regulator of the innate immune response and has been consistently implicated in chronic inflammation seen in PTSD and its comorbidities, including cardiovascular and autoimmune disorders. In PTSD, persistent NFκB activation may promote sustained release of proinflammatory cytokines (e.g., TNF-α, IL-6), creating a feed-forward loop that exacerbates both psychological and physical symptoms [16]. Thus, our findings linking these miRNA-target networks to PTSD symptom trajectories strengthen existing models of PTSD pathophysiology that emphasize neuroimmune and stress-signaling dysregulation.

This analysis identified key miRNAs exhibiting dynamic changes over time in response to the physical and psychological stressors experienced during and after deployment. We observed significant upregulation of three miRNA genes—miR-182-5p, miR-9-5p, and miR-204-5p—in individuals with PTSD compared to individuals without PTSD. These miRNAs are implicated in critical biological processes, including cell cycle regulation, cell migration, inflammation, transcriptional control, and neuronal circuitry, highlighting their potential roles in PTSD pathophysiology.

miR-204-5p has been shown to inhibit BDNF expression and affect various cellular processes [17]. In one prior study, knocking down miR-204-5p reversed the effect of corticosterone and flutamide on BDNF mRNA, suggesting that miR-204-5p plays a role in mediating the effects of androgen receptor and stress on BDNF expression. Another study observed that chronic social defeat stress (CSDS) can upregulate miR-204-5p in the hippocampus of mice and that its overexpression can worsen depressive-like behaviors [18]. Similarly, knockdown of miR-182-5p in the CSDS mouse model alleviated depression-like behaviors and impaired neurogenesis of CSDS-induced mice [19]. The mechanism may involve the Akt/GSK3β/CREB signaling pathway, which is crucial for neuronal survival, growth, and plasticity.

BDNF plays a crucial role in various neurological and psychiatric disorders. It is essential for the development of the central nervous system and neuronal plasticity. BDNF activates CREB through TrkB-mediated signaling pathways, and CREB, in turn, promotes BDNF transcription, regulating neuronal survival, synaptic plasticity, and memory. A systematic review found that PTSD patients had increased serum BDNF levels compared to healthy controls [20]. Our prior epigenetic research also identified the CREB–BDNF signaling pathway in PTSD cohorts [21]. In a longitudinal genome-wide methylation study, we identified that alterations in this pathway serve as convergent markers predicting both symptom severity and therapeutic outcomes in PTSD patients undergoing prolonged exposure therapy, with or without hydrocortisone augmentation [21]. Additionally, epigenetic biotypes of PTSD revealed distinct DNA methylation profiles associated with different PTSD subtypes, further implicating dysregulation in the dopamine-PKA-CREB and GABA-PKC-CREB signaling pathways [22]. 

Among the most striking findings, miR-9-5p also emerged as a key player, orchestrating the brain’s response to stress and medication with far-reaching implications. Research has shown that miR-9-5p rescues stress-induced dendritic shortening in hippocampal neurons, a critical mechanism underlying ketamine’s rapid antidepressant effects—suggesting a potential therapeutic linkage [23]. Beyond its role in neuroplasticity, miR-9-5p also modulates inflammatory responses, as demonstrated in traumatic brain injury studies where its upregulation mitigated blood-brain barrier damage and neuroinflammation. This protective effect is mediated through activation of the Hedgehog pathway and suppression of the NF-κB/MMP-9 pathway, suggesting that miR-9-5p could dampen the chronic inflammation often linked to PTSD [24].

Our analysis further uncovered four miRNAs—miR-185-5p, miR-194-5p, miR-199a-5p, and miR-133a-3p—that may signal predisposition to PTSD, offering a window into early risk identification. Notably, miR-133a-3p has been implicated in various neurological and psychiatric disorders, including depression, underscoring its relevance to mental health [25]. While miR-185-5p, miR-194-5p, and miR-199a-5p are less frequently associated with psychological conditions, they could play roles in PTSD-related comorbidities such as cardiometabolic [26] or inflammatory disorders [27], warranting further exploration. These pre-deployment biomarkers could aid in risk stratification before exposure to high-stress environments, such as military deployment, and support the development of preventive interventions.

However, not all miRNA patterns translated seamlessly from preclinical models to humans. Several miRNAs, including miR-144-3p [8], miR-142-5p [8], and miR-153-3p [9], showed dysregulation in brain regions of mouse or rat models of stress and trauma, but these changes were not statistically significant in the human blood-based study. This discrepancy may stem from species-specific differences in miRNA regulation or the distinct profiles of miRNAs in brain tissue versus peripheral blood, highlighting the challenges of translating animal findings to human PTSD research.

In addition to identifying trajectory-specific and PTSD-associated miRNAs, our analysis revealed significant sex differences in miRNA expression. Notably, all three differentially expressed miRNAs—miR-182-5p, miR-204-5p, and miR-9-5p—exhibited lower expression in females compared to males across the cohort. These findings suggest a potential sex-based regulatory mechanism contributing to PTSD vulnerability and symptom progression. Interestingly, among the pre-deployment biomarkers identified for PTSD risk, miR-194-5p also showed significantly higher expression in females. Given existing literature suggesting that females may be more susceptible to PTSD and that sex hormones influence epigenetic regulation, these sex-specific miRNA signatures may provide insight into underlying biological differences in PTSD susceptibility and resilience.

From a translational perspective, the identification of trajectory-linked miRNA signatures opens new avenues for clinical applications. First, miRNAs measurable in peripheral blood could serve as accessible biomarkers for early detection of PTSD risk, enabling proactive monitoring and personalized treatment planning. Second, their dynamic expression patterns across timepoints support their utility in tracking treatment response or recovery status in clinical trials. Third, the miRNA-target pathways identified here—such as BDNF–CREB, Notch, and NFκB signaling—highlight potential therapeutic targets for modulating neuroplasticity, stress resilience, and immune activation. Several candidate miRNAs (e.g., miR-9-5p, miR-204-5p) have already shown promise in preclinical models and could be explored further in pharmacological or gene therapy contexts. Nonetheless, translating these findings to clinical practice will require further validation. Importantly, we are currently analyzing the Fort Campbell cohort, an independent prospective PTSD dataset, to validate the diagnostic and prognostic utility of these miRNAs.

This study has several limitations that warrant consideration. First, although quality control measures confirmed miRNA stability in most samples, minor degradation over time cannot be fully excluded. This could potentially reduce the sensitivity for detecting low-abundance miRNAs, though our focus on differentially expressed miRNAs with high logCPM values (e.g., >6, Table 1) likely minimizes this impact. Second, the reliance on self-reported PTSD symptoms introduces potential subjective biases. However, previous studies using questionnaire instruments for PTSD have shown good agreement with medical diagnosis. Moreover, in some cases military personnel avoid seeking healthcare for mental disorders, which would likely lead to underreporting of PTSD using medical diagnoses [3]. Third, as miRNA profiles were analyzed in serum samples, inferences on their relevance to brain-specific changes associated with PTSD are indirect. The lack of uniformly available clinical covariates, such as comorbid conditions (e.g., diabetes, hypertension) and medication history, limited our ability to assess their potential influence on the observed molecular findings. The final limitation is the absence of an independent validation cohort to confirm the observed miRNA associations. We are currently analyzing data from the Fort Campbell cohort, an independent prospective PTSD study, to validate these findings as part of our future work.

## 4. Materials and Methods

### 4.1. Study Population

The Millennium Cohort Study is a comprehensive, long-term military prospective study initiated in 2001 to evaluate the health of military personnel over time [28]. Participants of the first enrollment phase were randomly selected from U.S. military personnel serving in October 2000, representing all service branches (Army, Navy, Air Force, Marine Corps) and components (active duty, Reserve, National Guard). Serum samples from 352 of these study participants, collected at all three follow-up waves close to the date of survey completion, were obtained from the Department of Defense Serum Repository (DoDSR). These samples were gathered separately from participation in the Millennium Cohort Study, typically as part of annual HIV testing or pre- and post-deployment collections. Participants were selected initially based on the availability of these serum samples, with eligibility requiring at least one deployment during the study period and completion of surveys at baseline (2001-03) and the follow-up waves (2004-05, 2007-08, or 2011-12). Subsequently, their survey data, including PTSD assessments using the PCL-C—a 17-item self-reported measure quantifying symptom severity over the past 30 days on a 5-point scale from “not at all” to “extremely”—were linked to the serum. The PCL-C scores were obtained in three survey cycles (2004-05, 2007-08, and 2011-13). Trauma experience was quantified using aggregate responses to five questions addressing personal trauma exposure. These questions captured exposure to witnessing death due to war or disaster, physical abuse, decomposing bodies, maimed individuals, or prisoners/refugees. Exposure frequency was categorized as none, once, or more than once, and scored on a scale of 1, 2, and 3, respectively. While not a direct replacement for the DRRI-2, this proxy captures key traumatic stressors (e.g., witnessing death, physical abuse) relevant to PTSD etiology, offering a reliable, simplified metric for statistical modeling.

Serum samples for 352 study participants were obtained from the DoD Serum Repository based on temporal proximity to the date of completion of the 2004, 2007, and 2011 surveys. The serum samples were transferred from DoDSR to WRAIR during 2015–2017 and stored at −80 °C until further analysis. Total RNA, including miRNA, was extracted from the serum samples using the miRNeasy Serum/Plasma Kit (Qiagen, Gaithersburg, MD, USA) according to the manufacturer’s protocol. Briefly, samples were thawed on ice, and 200 μL of serum was mixed with 1 mL of QIAzol Lysis Reagent(Qiagen, Gaithersburg, MD, USA). After incubation at room temperature for 5 min, 200 μL of chloroform was added, followed by vigorous shaking and incubation for an additional 3 min. Samples were then centrifuged at 12,000× *g* for 15 min at 4 °C. The aqueous phase was carefully transferred to a new tube, and 1.5 volumes of ethanol were added. The sample mixture was then applied to a RNeasy MinElute (Qiagen, Gaithersburg, MD, USA) spin column and washed with provided buffers. Finally, RNA was eluted in 14 μL of RNase-free water and stored at −80 °C until further processing.

### 4.2. miRNA Sequencing

Small RNA libraries were prepared using the TruSeq Small RNA Library Preparation Kit (Illumina, San Diego, CA, USA) following the manufacturer’s instructions. Briefly, 1 μg of total RNA was subjected to adapter ligation and reverse transcription, followed by PCR amplification. The amplified cDNA libraries were size-selected using a 6% polyacrylamide gel and purified using the QIAquick Gel Extraction Kit (Qiagen, Gaithersburg, MD, USA). The quality and concentration of the libraries were assessed using the Bioanalyzer 2100 system (Agilent Technologies, Santa Clara, CA, USA) and the Qubit Fluorometer (Invitrogen, Carlsbad, CA, USA), respectively. Most samples exhibited remain stable due to their small size and association with protective proteins (e.g., Argonaute), while larger serum-based RNAs are expected to undergo significant degradation. While long-term storage may lead to minor miRNA loss, prior studies suggest that miRNAs are robust under these conditions [29], and our normalization methods (TMM in edgeR) mitigate potential biases from variable RNA yield.

Sequencing was performed on the Illumina HiSeq 2500 platform (Illumina, San Diego, CA, USA), generating 50 bp single-end reads. Raw sequencing reads were imported into the CLC Genomics Workbench software (version 24.0, QIAGEN Bioinformatics) for processing. Adapter sequences were removed, and low-quality reads and reads shorter than 18 nucleotides were filtered out using the built-in small RNA analysis tools. Filtered reads were then mapped to the human reference genome (GRCh38) using the CLC Genomics Workbench’s small RNA alignment algorithm, which is optimized for aligning small RNA sequences. Known miRNA sequences from miRBase (release 22) were used for miRNA annotation.

CLC Genomics Workbench was used to obtain log counts per million (CPM) values for gene expression data, which accounts for sequencing depth and enables the comparison of expression levels between samples. First, low-count miRNAs were filtered out using a minimum expression threshold to improve statistical power. The remaining count data were then transformed using the voom function from the limma package v3.58.1, which models the mean-variance relationship and generates precision weights for downstream analysis. Batch effects from two experimental batches were subsequently removed using limma’s linear modeling approach with batch included as a covariate in the design matrix [30]. To validate the effectiveness of batch correction, we performed principal component analysis (PCA) before and after adjustment, which confirmed that no visual clustering by batch remained post-correction. Finally, we applied the trimmed mean of M-values (TMM) normalization method in the edgeR package v4.0.16 [31] to account for library size differences across samples prior to differential expression analysis.

### 4.3. Statistical Analysis

#### 4.3.1. Cluster Analysis

To examine the association between miRNA expression and PTSD symptoms, we employed a latent growth mixture model (LGMM) with PCL-C scores as the outcome and deployment history (prior deployment vs. no deployment), sex, and age as predictors. This model included a random intercept for waves nested within individuals to account for within-person correlations over time. We determined the optimal LGMM solution by evaluating a range of models with 1 to 5 latent classes, assessing model fit using Bayesian information criterion (BIC), alongside conceptual rationale and interpretability. Lower BIC value indicates better fit reflecting clearer class separation. To enhance the stability of the model, we performed 20 replications, and the cluster solution with the highest frequency of occurrence was selected.

PCL-C~deployment + sex + age +(wave|id)(1)

#### 4.3.2. Differential Analysis

DE analysis of miRNA profiles between groups (PTSD cases and healthy controls) was conducted using the edgeR package in R. A negative binomial model was applied to estimate the dispersion and log fold change between groups. The relationship between DE miRNAs and PTSD symptom severity was assessed using linear regression models, controlling for potential confounding factors such as age, sex, trauma experience, and waves. DE miRNAs were identified based on an FDR threshold of <0.05. The mRNA targets of the differentially expressed miRNAs were identified by querying miRTarBase. Further pathway analysis and functional enrichment of the mRNA targets were performed using Ingenuity Pathway Analysis (IPA) version 90348151. Overlapped targets were identified using Mienturnet 3.4.4, a web tool for microRNA-target enrichment analysis and network visualization.

DE analysis of miRNA profiles between PTSD cases and healthy controls was performed using the edgeR package in R [32]. We applied a negative binomial generalized linear model to estimate dispersion and log fold changes in miRNA expression between the groups, accounting for biological variability. DE miRNAs were identified using an FDR-adjusted *p*-value threshold of <0.05. The relationship between these DE miRNAs and PTSD symptom severity, measured by the PCL-C scores, was evaluated using linear regression models, adjusting for potential confounding factors, including age, sex, trauma experience (assessed via aggregate responses to trauma exposure questions, as described previously), and survey waves (baseline and follow-up cycles). mRNA targets of the DE miRNAs were predicted and validated by querying the miRTarBase database [33], which compiles experimentally supported miRNA–target interactions. Further pathway analysis and functional enrichment of these mRNA targets were conducted using Ingenuity Pathway Analysis (IPA) software (version 90348151), identifying key biological pathways and molecular functions associated with PTSD. Overlapping miRNA targets and their interactions were visualized and analyzed using Mienturnet [34], a web-based tool for microRNA target enrichment analysis and network visualization, to elucidate network-level relationships.

## 5. Conclusions

Our findings provide important insights into the molecular changes that occur in response to the physical and psychological stressors experienced by service members. These results are consistent with what is known about the pathophysiology of mood disorders, regulation of synaptic plasticity in learning and memory, and changes in neural circuits and brain structures associated with PTSD. These results may inform future studies of new screening methods to identify symptoms of PTSD early, enabling early intervention to mitigate further negative impacts on the health and well-being of military personnel. Further studies are needed to confirm and extend these findings and to examine the resiliency and vulnerability factors for long-term outcomes associated with PTSD, including comorbidities.

## Figures and Tables

**Table 1:** Comparison of demographic and clinical variables between control and PTSD groups across three waves.

	Wave 1			Wave 2			Wave 3		
Variable	Control	PTSD	*p*-Value	Control	PTSD	*p*-Value	Control	PTSD	*p*-Value
*n*	283	67		220	130		157	195	
Sex = female (%)	97 (34.3%)	25 (37.3%)	0.744	78 (35.5%)	45 (34.6%)	0.966	61 (38.9%)	62 (31.8%)	0.205
Trauma exposure (mean (SD))	6.92 (2.48)	7.70 (2.82)	0.04	6.86 (2.39)	7.41 (2.79)	0.006	6.33 (2.29)	7.46 (2.88)	0.006
Age category (%)			0.471			0.072			0.445
- 17–24 years	70 (24.7%)	22 (32.8%)		14 (6.4%)	19 (14.6%)		0 (0.0%)	0 (0.0%)	
- 25–34 years	146 (51.6%)	28 (41.8%)		112 (50.9%)	56 (43.1%)		64 (40.8%)	74 (37.9%)	
- 35–44 years	64 (22.6%)	16 (23.9%)		75 (34.1%)	43 (33.1%)		74 (47.1%)	88 (45.1%)	
- Greater than 44 years	3 (1.1%)	1 (1.5%)		19 (8.6%)	12 (9.2%)		19 (12.1%)	33 (16.9%)	
PHQ (mean (SD))	31.74 (10.31)	48.90 (15.50)	<0.001	33.57 (11.28)	48.76 (14.85)	<0.001	21.91 (17.60)	35.32 (26.62)	<0.001
Cigarette pack/week (mean (SD))	1.84 (0.77)	1.97 (0.87)	0.248	1.81 (0.77)	1.98 (0.81)	0.069	1.88 (0.90)	2.07 (0.91)	0.076
Sleep (mean (SD))	6.43 (1.33)	5.93 (1.65)	0.020	6.22 (1.10)	5.85 (1.59)	0.016	6.39 (1.36)	5.84 (1.51)	<0.001
PCL (mean (SD))	20.25 (3.49)	43.22 (10.87)	<0.001	20.95 (3.96)	41.52 (10.02)	<0.001	21.61 (4.30)	45.25 (11.53)	<0.001

**Table 2:** Differentially expressed miRNAs between PTSD positives and negatives.

	PTSD vs. Control				Female vs. Male		
miRNA	logFC	logCPM	*p*-Value	FDR	logFC	*p*-Value	FDR
hsa-miR-182-5p	0.4341	10.9460	6.33 × 10^−7^	0.0002	−0.4488	1.42 × 10^−7^	3.90 × 10^−6^
hsa-miR-204-5p	0.5628	7.0284	1.30 × 10^−6^	0.0002	−0.6623	2.19 × 10^−8^	8.75 × 10^−7^
hsa-miR-9-5p	0.5634	6.1838	2.37 × 10^−5^	0.0022	−0.6993	1.07 × 10^−7^	3.28 × 10^−6^

**Table 3:** Comparison of pre-deployment miRNA expression profiles between individuals who developed PTSD after deployment and those who remained resilient.

	PTSD vs. Control				Female vs. Male		
miRNA	logFC	logCPM	*p*-Value	FDR	logFC	*p*-Value	FDR
hsa-miR-185-5p	−0.4523	6.4460	9.02 × 10^−5^	4.13 × 10^−3^	0.2834	0.016	0.0819
hsa-miR-194-5p	−0.5232	6.5525	2.34 × 10^−7^	4.81 × 10^−5^	0.4883	2.16 × 10^−6^	0.0001
hsa-miR-199a-5p	−0.5414	6.6457	3.50 × 10^−7^	4.81 × 10^−5^	−0.1039	0.3332	0.4724
hsa-miR-133a-3p	−0.9689	9.2582	1.98 × 10^−6^	1.81 × 10^−4^	−0.1321	0.5322	0.6623

## Data Availability

The datasets generated and/or analyzed during the current study are not publicly available due to personally identifiable information regulations, but may be made available on reasonable request by the Naval Health Research Center Institutional Review Board (contact phone +1-619-553-8400).

## Supplementary Materials

The following supporting information can be downloaded at: https://www.mdpi.com/article/10.3390/ijms26115195/s1.

## Abbreviations

The following abbreviations are used in this manuscript:

BDNF	Brain-derived neurotrophic factor
CREB	cAMP Response Element-Binding Protein
DoDSR	Department of Defense Serum Repository
FDR	False discovery rate
LGMM	Latent growth mixture model
NFkB	Nuclear factor kappa-light-chain-enhancer of activated B cells
PCL	PTSD Checklist
TGF-alpha	Transforming Growth Factor-alpha
PTSD	Post-traumatic stress disorder
