# Supplementary material for: Long-Term miRNA Changes Predicting Resiliency Factors of Post-Traumatic Stress Disorder in a Large Military Cohort—Millennium Cohort Study
Source: Int J Mol Sci. 2025 May 28;26(11):5195. doi: 10.3390/ijms26115195 (PMC12155150; doi:10.3390/ijms26115195)
Supplement: Supplementary file 1 [file ijms-26-05195-s001.zip › ijms-3637307-supplementary.pdf]

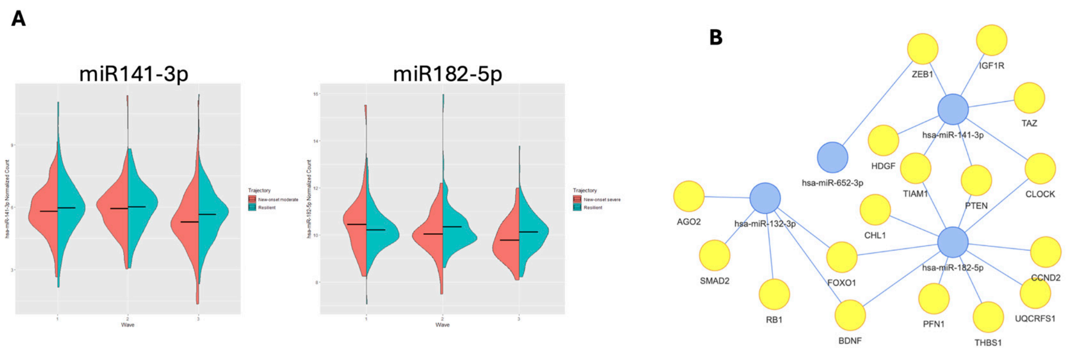

Figure S1. Differential miRNA Expression and Target Network in New-Onset vs. Resilient PTSD Trajectories. (A) Violin plots illustrate expression levels (logCPM) of two representative miRNAs (miR-141-3p, p=0.01; miR-182-5p, p=0.005) in new-onset PTSD groups (moderate, N=134; severe, N=35; red) versus the resilient group (N=132, blue) across three waves (2004–2011). New-onset groups show significant downregulation (e.g., miR-182-5p median logCPM ~8 vs. ~10 in resilient at Wave 2), with plot width reflecting expression density and medians marked by horizontal lines. (B) Network analysis maps all differentially expressed miRNAs to target genes, highlighting CLOCK (circadian regulation) and BDNF (neuroplasticity, p=0.008 for enrichment), key players in PTSD pathophysiology. Blue nodes denote miRNAs, yellow nodes represent targets, emphasizing neuronal survival pathways.
